# A bioenergetically‐active ploy (glycerol sebacate)‐based multiblock hydrogel improved diabetic wound healing through revitalizing mitochondrial metabolism

**DOI:** 10.1111/cpr.13613

**Published:** 2024-02-13

**Authors:** Xin Qi, Chenjun Liu, Jingyi Si, Bohao Yin, Jingjing Huang, Xin Wang, Jinghuan Huang, Hui Sun, Changfeng Zhu, Wei Zhang

**Affiliations:** ^1^ Department of Orthopedic Surgery Shanghai Sixth People's Hospital Affiliated to Shanghai Jiao Tong University School of Medicine Shanghai China; ^2^ Department of Orthopedic Surgery, Shanghai Institute of Microsurgery on Extremities Shanghai Sixth People's Hospital Affiliated to Shanghai Jiao Tong University School of Medicine Shanghai China; ^3^ Department of Gastroenterology and Hepatology, Zhongshan Hospital Fudan University Shanghai China

## Abstract

Diabetic wounds impose significant burdens on patients' quality of life and healthcare resources due to impaired healing potential. Factors like hyperglycemia, oxidative stress, impaired angiogenesis and excessive inflammation contribute to the delayed healing trajectory. Mounting evidence indicates a close association between impaired mitochondrial function and diabetic complications, including chronic wounds. Mitochondria are critical for providing energy essential to wound healing processes. However, mitochondrial dysfunction exacerbates other pathological factors, creating detrimental cycles that hinder healing. This study conducted correlation analysis using clinical specimens, revealing a positive correlation between mitochondrial dysfunction and oxidative stress, inflammatory response and impaired angiogenesis in diabetic wounds. Restoring mitochondrial function becomes imperative for developing targeted therapies. Herein, we synthesized a biodegradable poly (glycerol sebacate)‐based multiblock hydrogel, named poly (glycerol sebacate)‐co‐poly (ethylene glycol)‐co‐poly (propylene glycol) (PEPGS), which can be degraded in vivo to release glycerol, a crucial component in cellular metabolism, including mitochondrial respiration. We demonstrate the potential of PEPGS‐based hydrogels to improve outcomes in diabetic wound healing by revitalizing mitochondrial metabolism. Furthermore, we investigate the underlying mechanism through proteomics analysis, unravelling the regulation of ATP and nicotinamide adenine dinucleotide metabolic processes, biosynthetic process and generation during mitochondrial metabolism. These findings highlight the therapeutic potential of PEPGS‐based hydrogels as advanced wound dressings for diabetic wound healing.

## Graphical abstract

There was excessive oxidative stress, inflammation and mitochondria dysfunction in diabetic patient samples. poly (glycerol sebacate)‐co‐poly (ethylene glycol)‐co‐poly (propylene glycol) (PEPGS) hydrogel was synthesized through a polycondensation reaction of PEG, PPG, sebacic acid and Acr‐PEPGS. The implantation of PEPGS hydrogel promotes the healing of diabetic rats wounds by revitalizing mitochondrial metabolism.

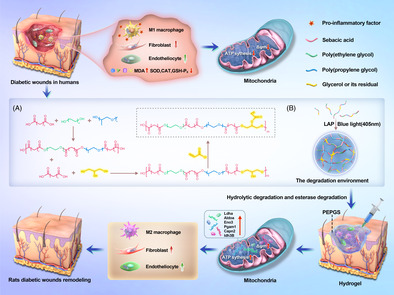

## 1 INTRODUCTION

Diabetic wounds, commonly known as chronic foot ulcers, present significant challenges in terms of impaired healing potential and increased risk of complications, posing significant burdens on patients' quality of life and healthcare resources worldwide.^1^, ^2^ The delayed healing trajectory of these wounds is primarily attributed to the intricate interplay of multifaceted physiological and pathological factors associated with diabetes mellitus, including hyperglycemia, oxidative stress, impaired angiogenesis, and excessive inflammation.^3^, ^4^, ^5^, ^6^, ^7^, ^8^, ^9^ It is noteworthy that these factors often coexist and interplay, creating a vicious cycle that contributes to the development and chronicity of diabetic wounds.^3^, ^10^, ^11^ Addressing these underlying mechanisms is crucial for designing effective strategies to manage diabetic wounds and preventing complications.

Emerging evidence indicates a close association between impaired mitochondrial function and diabetic complications, including chronic wounds.^3^, ^12^, ^13^, ^14^, ^15^, ^16^, ^17^ Wound healing is an energy‐demanding process that relies on various cellular processes, including cell migration, proliferation, collagen synthesis, immune response, and angiogenesis.^18^, ^19^ The importance of energy in wound healing cannot be overstated, as adequate energy supply plays a vital role in supporting these processes. Mitochondria, often referred to as the “powerhouses” of cells, play a critical role in providing the necessary energy for this complex healing process.^20^ In individuals with diabetes, mitochondrial dysfunction is a common occurrence due to chronic hyperglycemia, insulin resistance, and oxidative stress.^3^ Moreover, mitochondrial dysfunction often interacts bidirectionally with other pathological factors associated with diabetic wounds, exacerbating detrimental cycles that negatively impact cellular functions. For example, mitochondrial dysfunction can cause increased production of reactive oxygen species (ROS), while elevated levels of ROS can contribute to further mitochondrial damage.^3^ Mitochondrial dysfunction can contribute to impaired angiogenesis by disrupting endothelial cell metabolism and function, while inadequate angiogenesis can conversely lead to mitochondrial dysfunction due to insufficient oxygen and nutrient supply.^11^, ^21^ Therefore, it is reasonable to believe that mitochondrial dysfunction acts as a key driver and pathogenic hub in the chronicity of diabetic wounds. In this study, we conducted correlation analysis using clinical specimens, which revealed a positive correlation between mitochondrial dysfunction and oxidative stress, inflammatory response, and impaired angiogenesis in diabetic wounds.

Understanding the impact of mitochondrial dysfunction on diabetic wound healing is crucial for developing targeted therapeutic approaches aimed at restoring mitochondrial function and improving outcomes for individuals with diabetes. A few recent studies have provided new perspectives on restoring mitochondrial function to facilitate diabetic wound repair. For instance, Deng et al.^22^ investigated a double‐network hydrogel that exhibited antibacterial effects and maintained mitochondrial function for full‐thickness diabetic wound healing. Another study employed self‐assembled valsartan nano‐filaments to modulate mitochondrial energetics, resulting in faster healing in diabetic rat wounds.^23^ Xu et al.^15^ synthesized a thermosensitive hydrogel incorporating Prussian blue nanoparticles, which promoted diabetic wound healing via restoring mitochondrial function and scavenging ROS. Supporting mitochondrial function through targeted therapies and appropriate nutrition may enhance the efficiency and effectiveness of the diabetic wound healing process.

Hydrogels have emerged as promising biomaterials for wound healing applications due to their tunable mechanical properties and exceptional biocompatibility.^24^, ^25^, ^26^, ^27^, ^28^, ^29^ These three‐dimensional networks of hydrophilic polymers create a moist environment that mimics physiological conditions necessary for optimal wound healing. Hydrogels enable vital functions such as gas exchange, facilitating the diffusion of nutrients, removing excess exudate and protecting the wound from external contaminants. The unique properties of hydrogels collectively contribute to the promotion of an ideal wound‐healing environment. To optimize mitochondrial metabolism for wound healing, we focused on biodegradable poly (glycerol sebacate) (PGS) and its derivatives‐based hydrogels.^30^, ^31^, ^32^, ^33^, ^34^, ^35^, ^36^ Glycerol, one of the hydrolysis products of these polymers in vivo, is a naturally occurring sugar alcohol that cells can utilize as an energy source through its conversion to glycerol‐3‐phosphate (G3P).^37^ G3P enters the glycolysis pathway and eventually feeds into the tricarboxylic acid cycle (TCA or Krebs cycle), where it generates ATP through oxidative phosphorylation (OXPHOS) in mitochondria. Glycerol also contributes to maintaining cellular redox balance through participation in the G3P shuttle system, which transfers reducing equivalents from nicotinamide adenine dinucleotide into the mitochondrial electron transport chain, thus generating ATP and maintaining the redox state of mitochondria.^38^, ^39^ Herein, we developed a bioenergetically‐active PGS‐based multiblock hydrogel, known as poly (glycerol sebacate)‐co‐poly (ethylene glycol)‐co‐poly (propylene glycol) (PEPGS), with improved mechanical properties. Subsequently, we explored the wound‐healing applications of this formulation as a wound dressing. In vivo experiments demonstrated that the PEPGS hydrogel promoted diabetic wound healing by augmenting angiogenesis, fibrogenesis and reepithelization through stimulating energy production. Emerging evidence has highlighted that cellular metabolism is tightly linked to immune cell regulation of the activation phenotype and function.^40^, ^41^ Consequently, it was revealed that PEPGS hydrogel maintained ΔΨm, reduced ROS level, improved ATP synthesis and accelerated macrophage polarization to the M2 phenotype in the immune compartment. Proteomics analysis also exhibited the effects of PEPGS hydrogel on restoring mitochondrial metabolism during the process of diabetic wound healing.

## 2 MATERIALS AND METHODS

### 2.1 Macrophage polarization, immunohistochemical staining, oxidative stress detection and ATP production of wounds in the human body

Nondiabetic wound tissues (control group) were obtained from the wound edges of patients who underwent second‐stage (3 days) amputation (patients who selected a second‐stage amputation to save other limbs after undergoing severe limb trauma) (*n* = 3). Chronic diabetic wound tissues were obtained from the wound edges of patients who underwent a diabetic foot amputation (*n* = 3). When debridement was performed, the necrotic tissue was removed until skin tissue with an adequate blood supply was reached. We collected the trimmed edge of the skin tissue with an adequate blood supply for investigation. All patients were treated at the same institution.

To analyse the types of macrophages, skin wound‐edge samples in paraffin blocks were sectioned and treated with EDTA repair solution (pH 9.0, shanghaiwknowbio‐techo, Shanghai, China) for antigen retrieval. To test the specificities of inducible nitric oxide synthase (iNOS) for pro‐inflammatory macrophages (M1 macrophages) and CD206 for anti‐inflammatory macrophages (M2 macrophages), some tissue sections were incubated with iNOS and CD206, then the corresponding secondary antibody was added. Mouse monoclonal anti‐iNOS antibody (1:500, Abcam, Cambridge, UK) for M1 macrophages and rabbit polyclonal anti‐CD206 antibody (1:800, Servicebio, Wuhan, China) for M2 macrophages. Secondary antibodies of Cy3‐conjugated goat anti‐mouse IgG (Servicebio, Wuhan, China) and FITC‐conjugated donkey anti‐goat IgG (Servicebio, Wuhan, China) were used after the primary antibodies.

Immunohistochemistry of angiogenic marker CD31 (1:2000, Abcam, Cambridge, UK) was used to analyse impaired angiogenesis in diabetic wounds, the sections were rehydrated, blocked with 3% BSA and incubated with a primary antibody at 4°C overnight. After rehydration, sections were incubated with primary antibodies at 4°C overnight. Next, sections were counterstained with haematoxylin and analysed by light microscopy (Leica, Weztlar, Germany).

Then, ELISA kits were used to measure the malondialdehyde (MDA), superoxide dismutase (SOD), catalase (CAT) and glutathione peroxidase (GSH‐Px) concentrations in accordance with standard instructions (Njjcbio, Nanjing, China). After obtaining tissue lysates, the ATP levels in the tissue were determined luminometrically using an ATP assay reagent (Beyotime Institute of Biotechnology, Jiangsu, China) according to the manufacturer's protocol. The resulting luminescence was measured using a Varioskan LUX multimode plate reader (Thermo Scientific, Waltham, USA).

### 2.2 Preparation of PEPGS hydrogels

#### 2.2.1 Materials

Glycerol, sebacic acid, PEG (Mn = 1000), triethylamineand acryloyl chloride were purchased from Aladdin Chemistry Co., Ltd., (Shanghai, China).

#### 2.2.2 Preparation of PEPGS pre‐polymer

A polycondensation reaction of PEG, PPG and sebacic acid was carried out to obtain a linear prepolymer chain and to avoid any crosslinking. Here the molar ratio of PEG and PPG was 1:0, 4:1, 3:2, 1:1 and 0:1. The ratio of alcohol to acid was 1:2. Then, glycerol and sebacic acid were added to obtain a block copolymer of PEPGS (pre‐polymer) with a 30% content of alcohol segments.

#### 2.2.3 Synthesis of Acr‐PEPGS

Forty gram of PEPGS pre‐polymer was dissolved in dichloromethane. 7.9 mL of triethylamine was dripped under argon in an ice bath. Then 5.8 mL of acryloyl chloride was added to the mixed solution at 0°C and stirred for 48 h. After the reaction, the liquid was filtered, settled with ether, dialysed by dialysis bag and freeze‐dried for subsequent use.

#### 2.2.4 Chemical characterization

Fourier transform infrared spectra (Nicolet 5700, Thermo Fisher Scientific, Waltham, USA) of pre‐polymers were recorded in the range of 500–4000 cm^−1^. The molecular weight of PEGS and PEPGS pre‐polymers were determined by gel permeation chromatography (GPC, Shimadzu Prominence, Kyoto, Japan).

#### 2.2.5 Synthesis of PEPGS hydrogels

The water solubility of block copolymers containing different PEG/PPG ratios was investigated. Then, different concentrations of PEPGS were prepared for cross‐linking. Specifically, different amounts of Acr‐PEPGS were dissolved in aqueous solution containing the photoinitiator lithium phenyl‐2,4,6‐trimethylbenzoylphosphinate (LAP; 0.5 wt.%), crosslinked under blue light (405 nm), and liquidity was checked every 5 s until the polymer was moulded and cured, and the cross‐linking time was recorded. PEPGS was replaced by PEGS and was treated in the same way for cross‐linking preparation.

### 2.3 Characterization of PEPGS hydrogels

The surface morphology and microstructure of the PEPGS hydrogels were evaluated using a scanning electron microscope (Jeol Ltd, Tokyo, Japan).

To evaluate the mechanical strength of PEPGS hydrogels, a universal testing machine (Shimadzu Corp., Kyoto, Japan) was used for uniaxial tension and compression testing, equipped with a 20 N load cell. For uniaxial tensile testing, the cross‐linked sample was cut into dumbbell‐shaped pieces with a gauge length of 20 mm, a width of 5 mm and a thickness of about 1.2–1.9 mm. For compression testing, gel samples were prepared in a cylindrical shape, with a diameter of 10 cm and a thickness of 5–6 mm.

To evaluate the swelling behaviour of PEPGS hydrogel, samples of the same size were placed in PBS solution (pH = 7.4) and incubated at 37°C for 24 h, then removed for analysis of water absorption and swelling. The surface moisture was wiped off, then each sample was weighed, and the swelling ratio was calculated using the following formula:

$$\text{Swelling ratio}=\frac{\mathrm{wi}-w0}{w0},$$

where *wi* was the weight of the hydrogel after swelling, and *w*0 was the weight of the hydrogel before swelling.

To test the degradation properties of the hydrogels, the hydrogel samples were weighed (*w*2), then soaked in Tris–HCl buffer containing porcine liver esterase (Sigma‐Aldrich, St Louis, USA; 0.625 units per mg hydrogel) to better mimic the degradation behaviour in vivo. At different time‐points, the hydrogel was taken out and weighed as wi. The weight remaining was calculated using the following formula:

$$\text{Weight remaining}\;\left(\%\right)=\frac{\mathrm{wi}}{w2}\times 100\%.$$

#### 2.3.1 Biocompatibility

Human umbilical vein endothelial cells (HUVECs) and NIH 3T3 fibroblasts were obtained from the cell bank of the Chinese academy of sciences (Beijing, China).

The viability of the HUVECs and NIH 3T3 fibroblasts in the PEPGS hydrogels was investigated using the live/dead assay (YEASEN Shanghai Shengsheng Biotechnology Co., Ltd., Shanghai, China). Images were captured using a fluorescence microscope (Leica, Weztlar, Germany).

The proliferation of HUVECs and NIH 3T3 fibroblasts under different time points was evaluated using the Cell Counting Kit‐8 assay (Beyotime Institute of Biotechnology, Jiangsu, China); the absorbance of each well was measured at 450 nm using a spectrophotometric microplate reader (Bio‐Rad, Hercules, USA).

### 2.4 Cell experiments in vitro

We set up three groups: control group (culture medium alone), advanced glycation end‐products (AGEs) group (medium supplemented with 200 μg/mL AGEs) and AGEs + PEPGS group (medium supplemented with 200 μg/mL AGEs + 25 μL/cm^2^ PEPGS).

RAW 264.7 cells were obtained from the cell bank of the Chinese academy of sciences. JC‐1 (Beyotime Institute of Biotechnology, Jiangsu, China), Mito‐Tracker Red CMxRos (YEASEN Shanghai Shengsheng Biotechnology Co., Ltd., Shanghai, China) and a ROS Assay Kit (Beyotime Institute of Biotechnology, Jiangsu, China) were used to evaluate the mitochondrial membrane potential and ROS production. The images were captured using a DMi8 fluorescence microscope (Leica, Wetzlar, Germany). The cellular ATP levels were determined luminometrically using an ATP assay reagent (Beyotime Institute of Biotechnology, Jiangsu, China).

Expression of the M1‐type macrophage marker CCR7 and the M2‐type marker CD206 were further detected by flow cytometry (BD Biosciences, Franklin Lakes, USA). Finally, the cells were analysed by FCM and the data were acquired using the corresponding software.

Furthermore, we also analysed the expression levels of macrophage polarization‐related markers iNOS, Arg‐1, IL‐6, TNF‐α, IL‐4, and IL‐10 by RT–qPCR. RAW 264.7 primer information is provided in Table S1.

Cell migration was observed using a Transwell assay. Briefly, 2 × 10^4^ NIH 3T3 fibroblasts or HUVECs were seeded into the upper chamber of a transwell plate (Corning, NY, USA); a culture medium was added to the lower chamber. After 48 h (24 h for HUVECs), the cells on the upper surface of the membrane were removed using cotton swabs. The cells on the lower surface were fixed and stained with 0.5% crystal violet, and the results were observed via optical microscopy (Leica, Wetzlar, Germany).

For the capillary‐like construction activity of the HUVECs experiment, PEPGS hydrogels were spreaded on the upper chamber of a transwell plate, cold ECM gel (Sigma, St. Louis, USA) was transferred into the lower chamber of a transwell plate at 200 μL/well. Then, a 200‐μL suspension of pre‐treated HUVECs (8 × 10^4^ cells) was overlaid on the ECM gel. Eight hours later, tube formation was observed via light microscopy (Leica, Wetzlar, Germany).

For vascular endothelial growth factor (VEGF) and type I collagen (Col I) immunofluorescence assays, 1 × 10^4^ HUVECs or NIH 3T3 fibroblasts were seeded into wells of a 24‐well plate and cultured for 48 h. The cells were fixed for 10 min. An anti‐VEGF antibody (1:100, Abcam, Cambridge, UK) or anti‐Col I antibody (1:1200, Servicebio, Wuhan, China) was added to each well and incubated with the cells at 37°C, overnight. Next day, a secondary antibody (ABclonal, Woburn, USA) was added and incubated with the cells at 37°C for 1 h. Next, to visualize the nuclei, the samples were stained with DAPI for 5 min (Beyotime Institute of Biotechnology, Jiangsu, China), and to visualize the cytoskeleton, the samples were stained with phalloidin for 40 min after which they were observed using a confocal laser scanning microscope (Leica, Wetzlar, Germany).

### 2.5 In vivo studies of PEPGS in chronic diabetic wound closure

Twenty‐four male Sprague–Dawley (SD) rats (8 weeks old, 250–300 g) were used in this study. Streptozotocin (STZ; Sigma, St. Louis, USA) was used to induce diabetes. STZ was dissolved in 0.1 M phosphate–citrate buffer and injected intraperitoneally into SD rats at a dose of 55 mg/kg. Blood samples were collected from SD rat tail veins and measured using a glucometer (Roche, Basel, Switzerland). Skin wounds were created once the blood glucose levels were >250 mg/mL, which indicated diabetes. After anaesthesia by intraperitoneal injection of 1 mL/kg of 3% pentobarbital sodium (Sigma, St. Louis, USA), and standardized full‐thickness skin wounds were generated (diameter = 2.0 cm). The rats were then randomly assigned to two different groups, which were treated with phosphate‐buffered saline (PBS, control group) and PEPGS (PEPGS group). PBS was injected around the wound at four injection sites and applied to the wound bed, then the wounds were covered with gauze. On days 0, 3, 7, and 14 after the procedure, the wounds were observed. Wound closure was calculated according to the following equation: $\text{wound closure}\;\left(\%\right)=\left(A_{O}-A_{t}\right)/A_{O}\times 100$, where *A*_O_ is the initial wound area, and *A*_t_ is the wound area on the observation days. The rats were sacrificed on days 7 or 14 after the procedure, and the wound tissues containing the wound bed and surrounding healthy skin were carefully collected. The wound tissue samples were fixed in formalin, subjected to dehydration in graded alcohol solutions and embedded in paraffin. Sections (5 μm) were generated and stained with haematoxylin–eosin and Masson's trichrome.

Immunofluorescence staining of macrophages and immunohistochemical staining of inflammatory markers were the same as wounds in the human body described above. Reagent information is as follows: rabbit polyclonal anti‐iNOS antibody (1:500, Abcam, Cambridge, UK), mouse polyclonal anti‐CD206 antibody (1:1800, Servicebio, Wuhan, China), secondary antibodies of Cy3 goat anti‐mouse IgG (1:1300, Servicebio, Wuhan, China), FITC‐conjugated donkey anti‐goat IgG (1:300, Servicebio, Wuhan, China), primary antibodies against IL‐6 (1:200, HUABIO, Woburn, USA) and TNF‐α (1:100, Proteintech, Rosemont, USA), secondary antibodies (Servicebio, Wuhan, China), CD31 (1:250, Abcam, Cambridge, UK), and alpha‐smooth muscle actin (α‐SMA; 1:500, Servicebio, Wuhan, China) immunofluorescence was used to estimate angiogenesis. The sections were rehydrated, blocked with 3% BSA and incubated with a primary antibody at 4°C overnight. The next day, Alexa Fluor 488‐ and Cy3‐conjugated secondary antibodies (Servicebio, Wuhan, China) were applied, and DAPI was used to stain the nuclei. Samples were observed by confocal laser microscopy (Leica, Wetzlar, Germany).

Microfil (Flow Tech, Carver, USA) was used to assess blood vessel regeneration. Briefly, the chest of each rat was opened using scissors. After ligating the bilateral pulmonary trunks, the inferior vena cava was punctured. Heparinized saline (100 mL) was perfused into the left ventricle through an angiocatheter, and 20 mL of Microfil was perfused at a rate of 2 mL/min into the left ventricle, then the ascending aorta was ligated. The rats were maintained at 4°C overnight to ensure polymerization. The samples were then scanned using micro‐CT at a resolution of 9 μm and images were reconstructed using 3D creator software. The blood vessel area was determined using the software.

Proteomics analysis was performed by a commercial company (Wayen Biotechnologies, Shenzhen, China). Briefly, samples were homogenized by a high‐speed cryogenic grinding machine, then the intermediary solution was transferred to a new EP tube. After protein quantification, dithiothreitol was added to 100 μg protein solution at a final concentrations of 10 mM. Iodoacetamide (dithiothreitol:iodoacetamide = 1:5) was added, and incubated in the dark for 40 min. Next, five times the volume of precipitation reagent was added, and the mixture was allowed to precipitate for 1 h at 4°C. Samples were then centrifuged at 13,000 rpm for 1 h, then 1 mL of 100% acetone was added to wash the precipitate, and the samples were centrifuged again at 13,000 rpm for 30 min. The precipitate was dried at room temperature for 10 min. The polypeptide was desalinated after centrifuging and drying by monospin desalination, then analysed using mass spectrometry after vacuum drying.

After analysis, the above residual protein samples were used to perform multiple reaction monitoring (MRM). Briefly, The target protein list was imported into skyline software to match the corresponding library, quantitative characteristics of target protein peptides were screen, then target peptides were detected based on the MRM pattern. Finally, the MRM raw data of all samples were imported into skyline quantitative statistical software. Fold change ≥1.2 and *p* value < 0.05 were considered to indicate differentially expressed proteins. To elucidate the protein–protein interaction networks and perform module analysis, differentially expressed molecules were analysed using the Search Tool for the Retrieval of Interacting Genes/Proteins database (available at https://string-db.org/). The interactions between proteins of the target genes were identified and the functional interactions were assessed using confidence scores. The interaction networks were then visualized using the cytoscape software.

### 2.6 Statistical analysis

All data were analysed by one‐way ANOVA followed by the Student–Newman–Keuls test and linear regression analysis using SPSS 26.0 software (IBM SPSS Statistics for Windows, Armonk, USA). The Kolmogorov–Smirnov test was used to analyse whether the data were normally distributed, and the test of homogeneity of variance was used to analyse whether the data were qualified for homogeneity of variance. All data are presented as the mean ± standard deviation (SD), and *p* < 0.05 was considered statistically significant.

## 3 RESULTS

### 3.1 Correlation analysis of normal and diabetic wounds between mitochondrial functions and oxidative stress, inflammatory response and impaired angiogenesis

**FIGURE 1:**
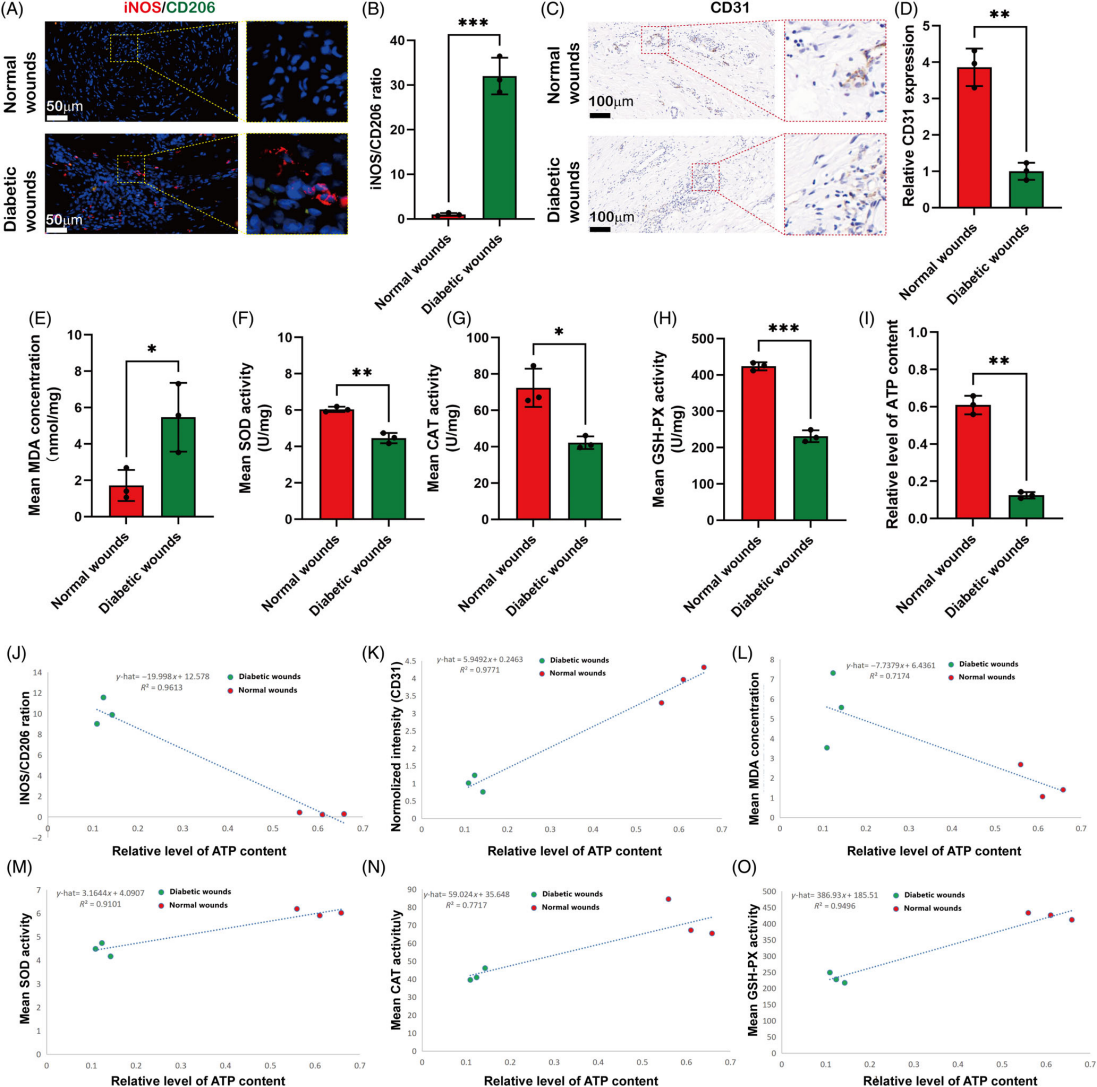
Comparative analyses of macrophage polarization, ATP production and oxidative stress between normal and diabetic wound tissue. (A) Representative immunofluorescence staining of inducible nitric oxide synthase (iNOS; M1 macrophage marker) and CD206 (M2 macrophage marker) in the wound bed; nuclei were stained with DAPI. (B) Quantitative analysis of iNOS/CD206 ratio. (C, D) Immunohistochemical staining of CD31 in the wound bed and quantitative analysis of angiogenesis data. (E) Quantitative analysis of the content of malondialdehyde (MDA). (F–H) Quantitative analysis of antioxidant enzyme system activity (superoxide dismutase [SOD], catalase [CAT], and glutathione peroxidase [GSH‐Px]). (I) Quantitative estimation of ATP level. (J–O) Linear regression analysis between levels of ATP production and oxidative stress, inflammatory response, and impaired angiogenesis. Data are presented as the means ± SD. *p < 0.05, **p < 0.01 and ***p < 0.001. *n* = 3 per group.

The results showed that the intensity of iNOS (red, M1 marker) staining in positive cells was significantly higher in the diabetic wound group (Figure 1A), and the iNOS/CD206 (green, M2 marker) ratio of the diabetic wound group (1.00 ± 0.33) was significantly lower compared with that of the control group (32.02 ± 4.09, Figure 1B, *p* < 0.001). The immunohistochemical staining of CD31 results revealed the numbers of newly formed and mature blood vessels in wounds of the different groups (Figure 1C). Qualitative results showed that the degree of impaired angiogenesis was significantly worse in diabetic wounds compared with normal wounds (Figure 1D, *p* < 0.01). The MDA levels were 1.72 ± 0.85 nmol/mg in the normal wound group but were significantly higher (5.64 ± 1.63 nmol/mg) in the diabetic wound group (Figure 1E, *p* < 0.05). Meanwhile, the mean levels of SOD, CAT and GSH‐Px were 6.04 ± 0.14 U/mg, 72.41 ± 10.54 U/mg, and 423.78 ± 10.89 U/mg, respectively, in the normal wound group. However, compared with the normal group, the mean levels of SOD (4.46 ± 0.28 U/mg), CAT (42.24 ± 3.44 U/mg), and GSH‐Px (231.40 ± 16.15 U/mg) were significantly reduced in the diabetic wound group (Figure 1F–H, *p* < 0.01, *p* < 0.05, *p* < 0.001). The results showed that the ATP production levels were significantly reduced in diabetic wounds (0.13 ± 0.02 μM) compared with the normal group (0.61 ± 0.05 μM; Figure 1I, *p* < 0.01). Therefore, we used linear regression analysis to identify correlations between mitochondria functions and oxidative stress, inflammatory response and impaired angiogenesis. The results suggest that mitochondrial dysfunction is positively correlated with oxidative stress, inflammatory response and impaired angiogenesis in diabetic wounds (Figure 1J–O).

### 3.2 Preparation and characterization of PEPGS hydrogels

**FIGURE 2:**
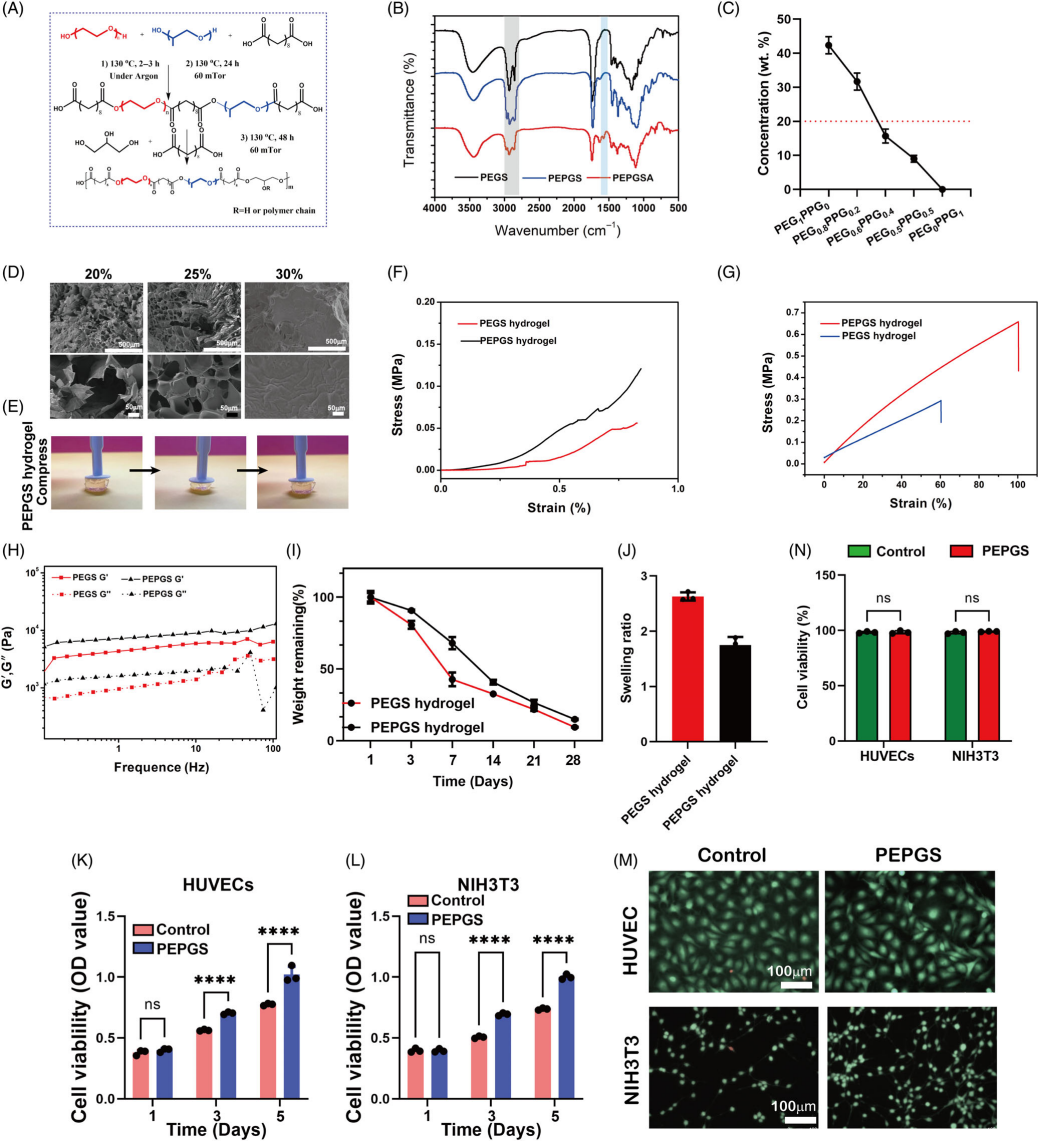
Fabrication and characterization of poly (glycerol sebacate)‐co‐poly (ethylene glycol)‐co‐poly (propylene glycol) (PEPGS) hydrogel. (A) Illustration of the synthetise of PEPGS prepolymer. (B) Fourier transform infrared spectra of the PEGS, PEPGS and PEPGSA polymers. (C) Water solubility of PEPGS polymer at different PEG and PPG ratios. (D) SEM images of PEPGS hydrogels at different polymer concentration. (E) Photographs of the hydrogels withstanding compression. (F) Stress–strain curves of hydrogels. (G) Tensile tests of different hydrogel. (H) Rheological studies over frequency used to assess mechanical property of the hydrogel. (I) Degradation curves of hydrogels. (J) Swelling ratio of the hydrogel. (K, L) Cell proliferation analysis of human umbilical vein endothelial cells (HUVECs) and NIH 3T3 on PEPGS hydrogel by Cell Counting Kit‐8 after incubating for 1, 3, and 5 days. (M, N) Live/Dead staining of HUVECs and NIH 3T3 at 24 h. Data are presented as the means ± SD. ns, no statistical significance. ****p < 0.0001. *n* = 3 per group.

To prepare the PEPGS hydrogel, PEPGS pre‐polymer and Acr‐PEPGS were synthesized (Figure 2A). Fourier transform‐infrared analysis showed the chemical structure of the polymers **(**Figure 2B **)**. A strong peak around 1730 cm^−1^ corresponding to an ester group and a broad absorption peak of hydroxyl groups at 3500 cm^−1^ (–OH) was observed in all polymers, Moreover, the characteristic absorption peak of a methyl group around 2240 cm^−1^ was absent from the PEPGS pre‐polymer and the Acr‐PEPGS polymer. The new characteristic absorption of the acrylate group at ∼1635 cm^−1^ indicated the introduction of acrylate functional groups in Acr‐PEPGS. The Mw of the prepolymer was analysed by GPC and showed that PEGS (11.39 kDa) and PEPGS (13.23 kDa) prepolymers had similar Mw. Due to the hydrophobicity of the chain end of the polypropylene glycol, we first investigated the solubility of the polymer with different contents of PEG and PPG **(**Figure 2C **)**. As the content of PPG increased, the water solubility of the polymer decreased, and the maximum solubility decreased from ca. 40% to ca. 1%. Since the minimum concentration required for subsequent crosslinking was 20%, a ratio of PEG to PPG of 4:1 was chosen for subsequent experiments. PEPGS hydrogels of different concentrations (20%, 25%, 30%) were prepared and measured. With increasing concentration, the cross‐linking became faster, but for all hydrogels, the gelation time was less than 10 s. Through SEM imaging (Figure 2D), it was observed that the pores within the gels of all three concentrations became smaller with increasing polymer concentration. When the concentration reached 30%, the hydrogel became denser. Consequently, a 25% concentration was chosen for gel preparation.

Through compression and tensile tests (Figure 2E–G), it was observed that the elastic modulus of the PEPGS hydrogel was significantly increased compared with the PEGS hydrogel under the same preparation conditions (concentration, crosslinking time, and initiator content), which proved that the introduction of a PPG chain end effectively enhanced the mechanical properties. Additionally, we examined the frequency‐dependent rheological behaviour using oscillation frequencies ranging from 0.1 to 100 Hz. As shown in Figure 2H, under the test frequency, the storage modulus (*G*′) of all hydrogels were consistently higher than the loss modulus modulus (*G*″), which indicates their elastic behaviour. At the same time, the mechanical properties of the PEPGS hydrogel exhibited a significant enhancement to the PEGS hydrogel.

An appropriate swelling rate plays an important role in maintaining the integrity and mechanical properties of a dressing. Therefore, the swelling property of the PEPGS hydrogel was investigated and compared with that of the PEGS hydrogel. The ideal biomaterial scaffold for tissue engineering should have a degradation rate matching the rate of new tissue regeneration. Both PEGS and PEPGS hydrogels were completely degraded in 30 days with esterase (Figure 2I), established by the hydrolysis of hydrogels. The swelling rate of PEPGS was lower than that of PEGS hydrogel (Figure 2J), which is related to the hydrophobicity of PPG.

### 3.3 Cytotoxicity assessment of the PEPGS hydrogels

Cell proliferation was analysed over durations of 1, 3, and 5 days. As shown in Figure 2K,L, the OD values increased with time in all groups, suggesting that the PEPGS hydrogel did not have a toxic effect on the cells. Moreover, from 3 days, the OD value in all PEPGS hydrogel groups was higher than in the control group (*p* < 0.0001), which indicated that PEPGS hydrogels enhanced cell proliferation activity. To directly observe cell viability, live/dead staining was carried out to evaluate cells cultured with PEPGS hydrogels. As expected, HUVECs and NIH 3T3 cells (Figure 2M,N) on the PEPGS hydrogels exhibited high viability at 24 h after seeding, and almost no dead cells were found.

### 3.4 PEPGS maintained ΔΨm, reduced ROS level, facilitated M2 macrophage polarization and improved ATP synthesis through enhanced mitochondrial metabolism

**FIGURE 3:**
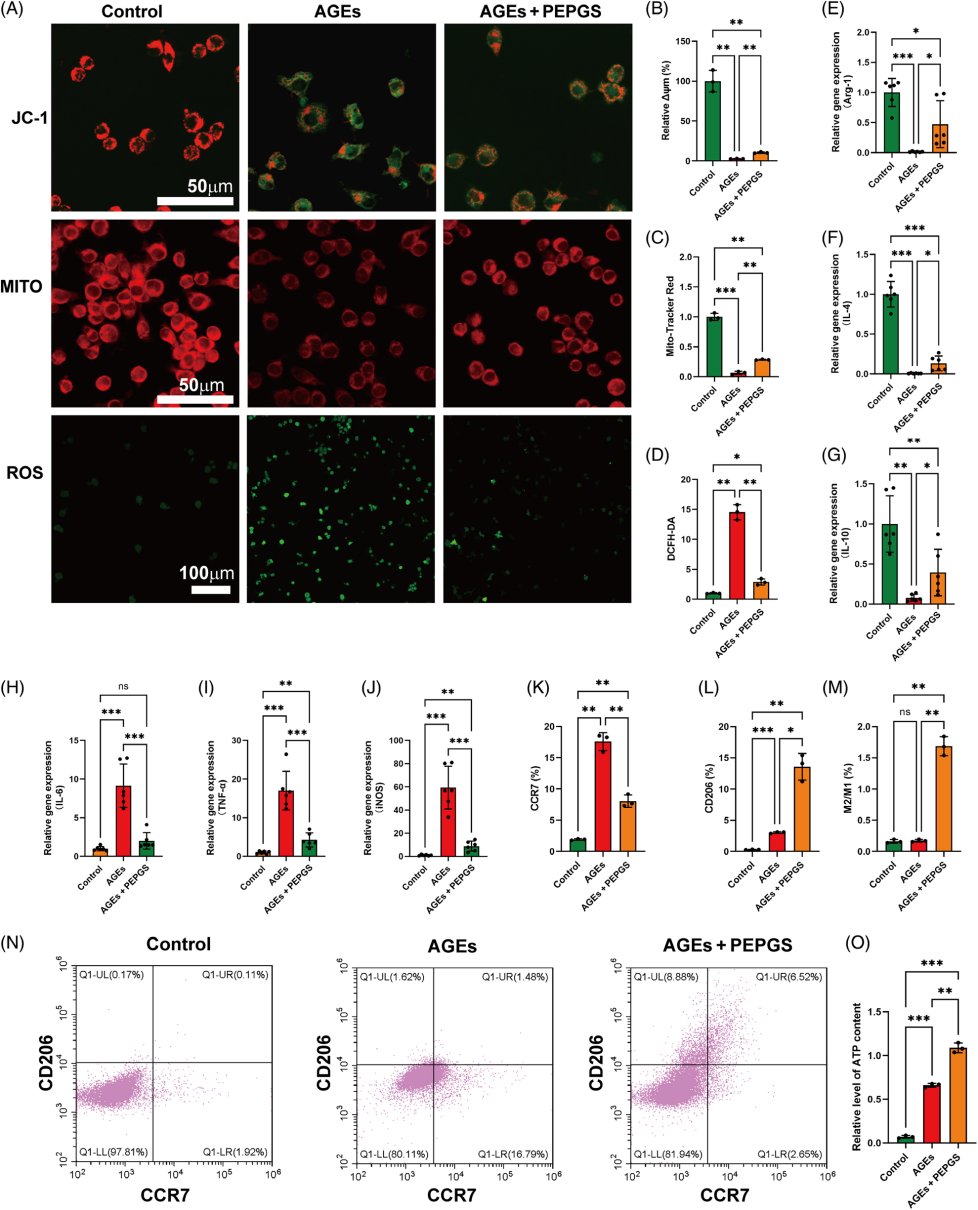
The effect of poly (glycerol sebacate)‐co‐poly (ethylene glycol)‐co‐poly (propylene glycol) (PEPGS) hydrogel on ΔΨm, reactive oxygen species (ROS) level, macrophages polarization and ATP synthesis. (A) Immunofluorescence staining of mitochondrial membrane potential (ΔΨm) detected by JC‐1 probe and Mitotracker indicator and intracellular ROS, respectively. (B–D) Quantitative analysis of the effect of PEPGS hydrogel on expression of JC‐1, MItotracker and ROS. (E–G) RT–qPCR results of M2 related‐maker: IL‐4, IL‐10, Arg‐1. (H–J) RT–qPCR results of M1 related‐maker: IL‐6, TNF‐ɑ, and inducible nitric oxide synthase (iNOS). (K–N) Polarization of RAW 264.7 cells analysed by flow cytometry. (O) Quantitative estimation of ATP level. Data are presented as the means ± SD. ns, no statistical significance. *p < 0.05, **p < 0.01 and ***p < 0.001. *n* = 3 per group. AGEs, advanced glycation end‐products.

In our research, RAW 264.7 cells were used as an experimental model of macrophages. Total ROS levels were analysed using DCFH‐DA reagents. The relative DCFH‐DA fluorescence intensity in the PEPGS group was significantly lower than in the AGEs group (Figure 3A,D), reflecting the fact that the ROS level in PEPGS was reduced. The immunofluorescence results showed notable JC‐1 monomers in the AGEs group, indicating a significant decrease in ΔΨm. Interestingly, the PEPGS group effectively stabilized the ΔΨm of mitochondria compared with the AGEs group (Figure 3A,B). Results of Mito‐Tracker Red CMXRos immunofluorescence were consistent with those obtained with the JC‐1 probe (Figure 3A,C).

The RT‐qPCR results presented M2 macrophage markers Arg‐1, IL‐4, and IL‐10 (secreted by M2 macrophages) were upregulated in the PEPGS group (Figure 3E–G, *p* < 0.05), suggesting a higher proportion of M2 macrophages. Additionally, RT–qPCR showed higher iNOS (M1 macrophage marker) expression and upregulated TNF‐α and IL‐6 expression in the AGEs group compared with the PEPGS group (Figure 3H–J, *p* < 0.001). To further explore how PEPGS regulates the polarization of RAW 264.7 cells, we examined the proportions of CCR7+ (M1 marker) and CD206+ (M2 marker) among the RAW 264.7 cells by flow cytometry (Figure 3K–N). Compared with the AGEs group (3.03 ± 0.14%), the PEPGS group (13.58 ± 2.13%) induced RAW 264.7 cells to transform into more M2 macrophages at 4 days (Figure 3L, *p* < 0.05). In addition, the M2/M1 ratio of the PEPGS group was significantly increased compared with that of the AGEs group (Figure 3M, *p* < 0.01). We then evaluated the effect of PEPGS on ATP levels, using a luciferase detection kit. It was found that the PEPGS successfully restored ATP production to some extent (AGEs + PEPGS vs AGEs, 1.09 ± 0.06 μM vs 0.66 ± 0.02 μM, Figure 3O, *p* < 0.01).

### 3.5 Effect of PEPGS hydrogels on HUVECs and NIH 3T3 fibroblasts under a replicated diabetic microenvironment in vitro

**FIGURE 4:**
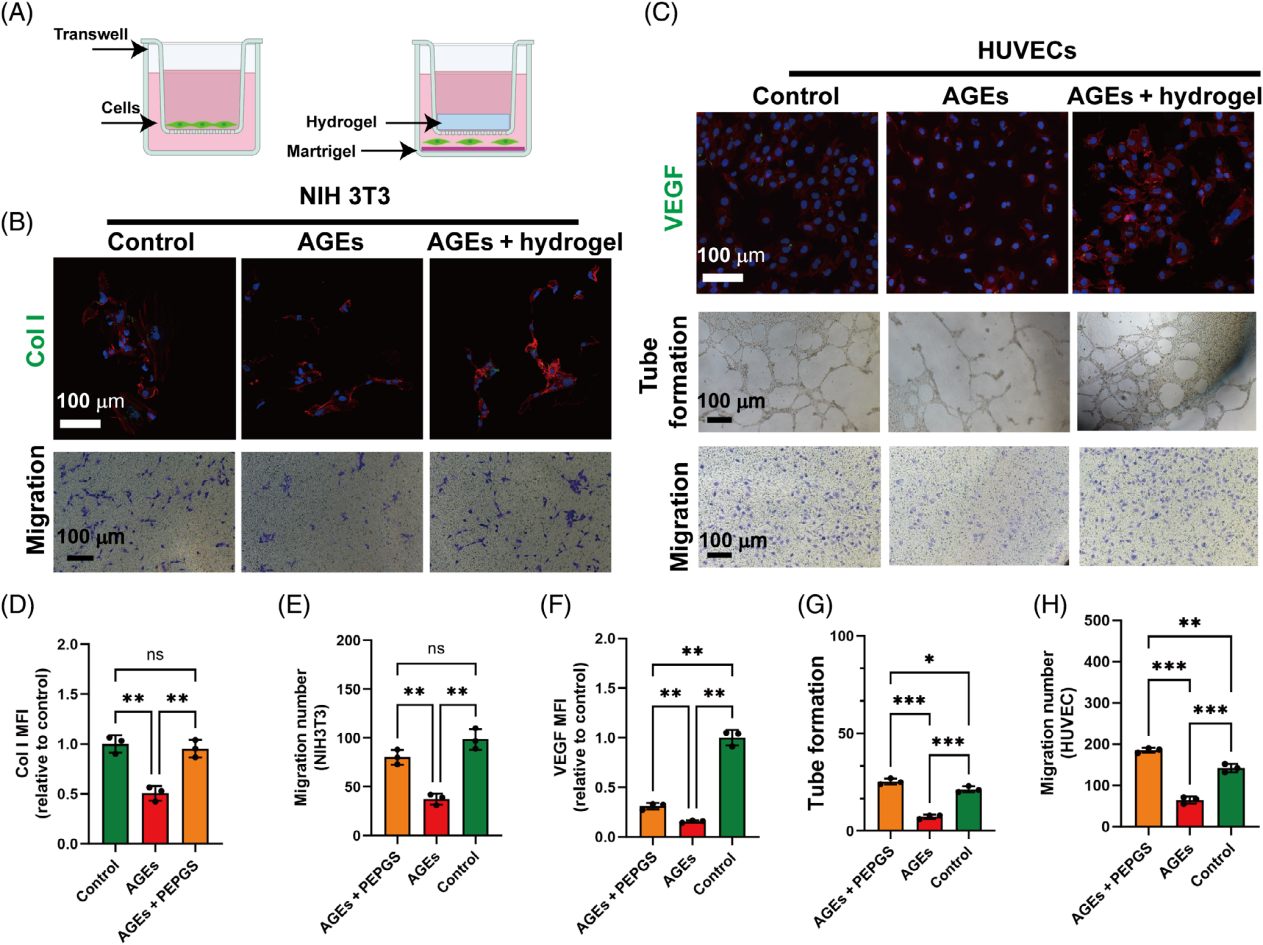
The effect of poly (glycerol sebacate)‐co‐poly (ethylene glycol)‐co‐poly (propylene glycol) (PEPGS) hydrogel on the human umbilical vein endothelial cells (HUVECs) and NIH 3T3 under a simulated diabetic microenvironment. (A) Schematic diagram of the cell migration and tube formation experiment. (B) Type I collagen (Col I) immunofluorescence in NIH 3T3 on PEPGS hydrogel (red represents the cell cytoskeleton; green represents Col I; blue represents nuclei); PEPGS hydrogel effects on NIH 3T3 cell migration. (C) Vascular endothelial growth factor (VEGF) immune‐ fluorescence in HUVECs on PEPGS hydrogel (red represents the cell cytoskeleton; green represents VEGF; blue represents nuclei); PEPGS hydrogel effects on capillary‐like structure formation; PEPGS hydrogel effects on NIH 3T3 cell migration. (D, E) Quantitative analysis of the effect of PEPGS hydrogel on Col I expression and cell migration of NIH 3T3 cells. (F–H) Quantitative analysis of the effect of PEPGS hydrogel on VEGF expression, migration and tube formation of HUVECs. Quantitative data on tube formation (G) was presented from statistics on the number of vascular rings in the field of view in (C, Tube formation). Data are presented as the means ± SD. ns, no statistical significance. *p < 0.05, **p < 0.01 and ***p < 0.001. *n* = 3 per group.

In this study, cell cultures were supplemented with AGEs as an inducer to mimic the diabetic microenvironment. Cultures fed with fresh medium alone served as the control group. We first analysed cell migration (Figure 4A–C) in HUVECs and NIH 3T3 fibroblasts in vitro. Transwell assays showed that PEPGS hydrogels significantly promoted the migration of HUVECs (Figure 4G) and NIH 3T3 fibroblasts (Figure 4E) compared with the AGEs group. Then, tube formation assays using HUVECs were used to determine the pro‐angiogenic potential of PEPGS hydrogels. After incubating on Matrigel substratum for 8 h, HUVECs incubated with AGEs formed sparse or even incomplete tube networks (Figure 4C), whereas those on PEPGS hydrogels exhibited significantly improved tube‐forming ability in the presence of AGEs (Figure 4H). To further investigate the effect of PEPGS hydrogels on the differentiation potential of HUVECs and NIH 3T3 fibroblasts in a diabetic microenvironment in vitro, the expression of two factors Col I and VEGF crucial in angiogenesis and fibrogenesis were observed by immunofluorescence staining (Figure 4B,C). The results showed that PEPGS hydrogels positively regulated the fibrogenic and angiogenic abilities of both cell types compared with the AGEs group (Figure 4D,F, *p* < 0.01).

### 3.6 PEPGS hydrogels promoted the healing of diabetic wounds in vivo due to their pro‐angiogenic, pro‐fibrogenic and immunoregulatory activities

**FIGURE 5:**
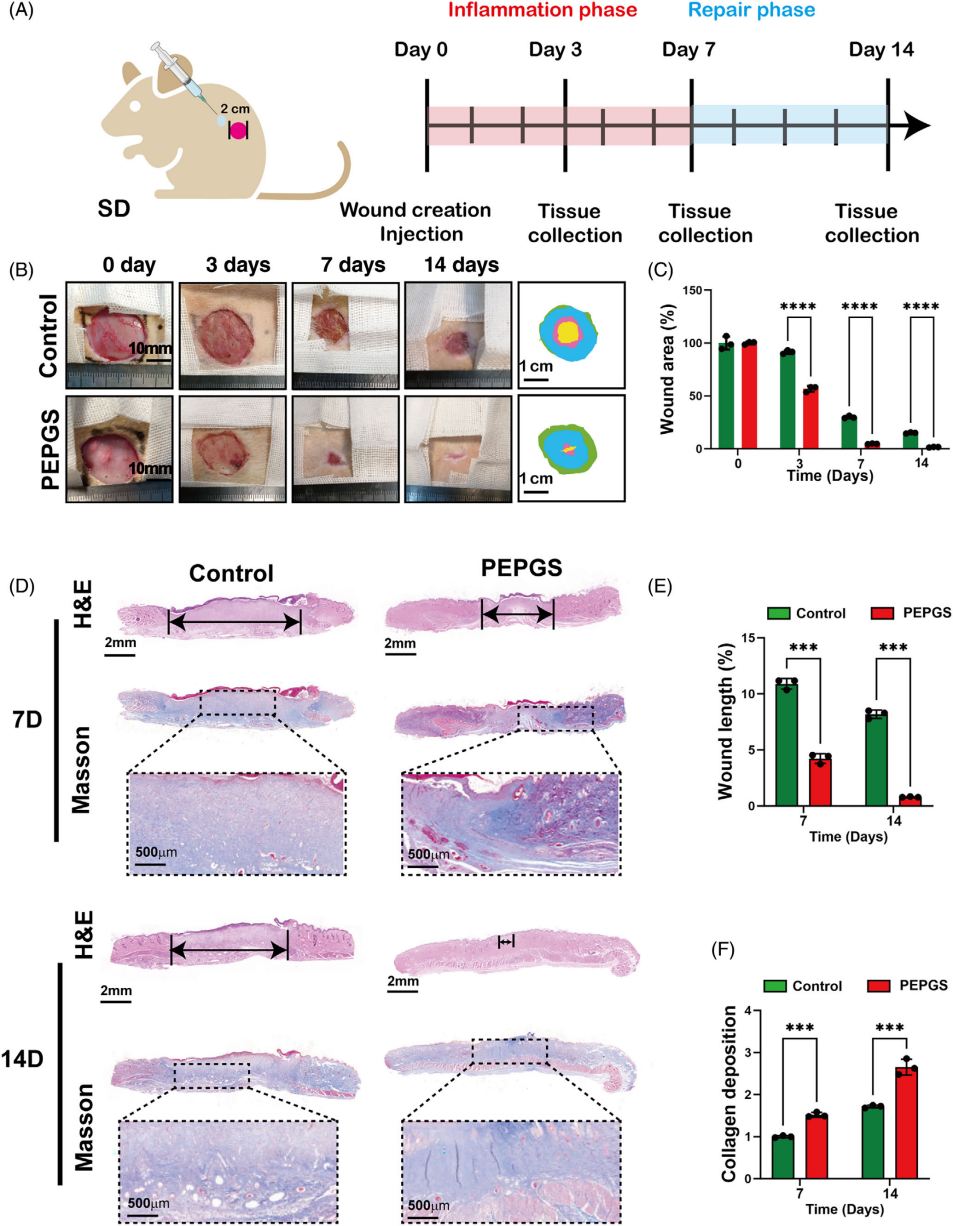
Diabetic wound closure after treatment with poly (glycerol sebacate)‐co‐poly (ethylene glycol)‐co‐poly (propylene glycol) (PEPGS) hydrogel. (A) Schematic diagram of model wound creation and the healing process of diabetic wounds. (B, C) General observation and healing rate of diabetic wounds under different time points. (D–F) Haematoxylin–eosin (H&E) staining of healing wounds and Masson's trichrome staining of collagen deposition in wounds. ***p < 0.001 and ****p < 0.0001. *n* = 3 per group.

In vivo, the wound‐healing process and rate were analysed. Angiogenesis, fibrogenesis and immunoregulation are key processes in wound healing that were observed in our study. No significant adverse reactions or complications were observed throughout the duration of the experiment. Wound closure progressed from 0 to 14 days and was recorded and quantified using digital images (Figure 5A–C). The results showed that the wounds of the PEPGS group were substantially smaller than those of the control group on days 3 and 7. By 14 days, the wounds of the PEPGS group were almost closed, while those of the control group remained patent.

Haematoxylin–eosin staining showed re‐epithelization 7 and 14 days after surgery (Figure 5D). The results showed that the length of neoepithelium was significantly greater and wound closure was significantly better in the PEPGS group than in the control group (Figure 5D,E, *p* < 0.001). The wounds treated with the PEPGS were almost closed, and neoepithelium covered the entire wound, while in the control group, the wounds were about 50% covered. Masson's trichrome staining (Figure 5D) revealed differences in collagen deposition and composition between the different groups during wound healing. Compared with wounds of the control group, the wounds treated with PEPGS had more collagen deposition and thicker wavy collagen fibres (Figure 5F, *p* < 0.001), and the arrangement of wavy collagen fibres was similar to that in normal skin.

**FIGURE 6:**
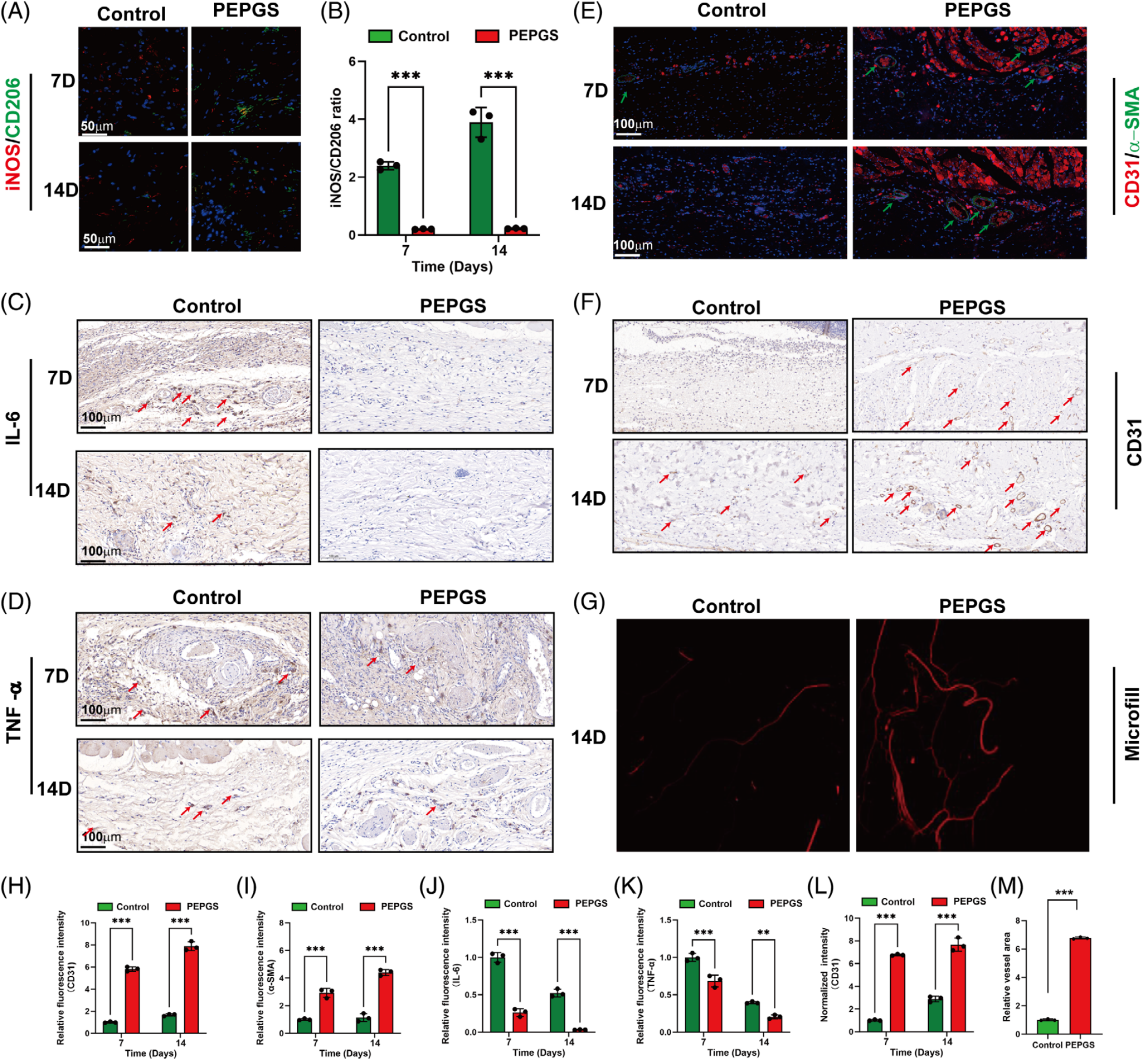
Histological assessment after treatment with poly (glycerol sebacate)‐co‐poly (ethylene glycol)‐co‐poly (propylene glycol) (PEPGS) hydrogel during diabetic wound healing. (A, B) Representative immunofluorescence staining and quantitative analysis of inducible nitric oxide synthase (iNOS; red, M1 macrophage marker) and CD206 (green, M2 macrophage marker) in wound bed at 7 days and 14 days, the nuclei were stained with DAPI (blue). (C, D) Representative images of IL‐6 (red arrow) and TNF‐α (red arrow) taken at 7 days and 14 days. (E–G) Representative image of immunofluorescence (CD31/α‐SMA [green arrow]), immunohistochemical staining (CD31, red arrow) and 3D‐reconstructed image of newly‐formed capillaries. (H, I, L, M) Quantitative analysis of angiogenesis data. (J, K) Quantitative analysis of IL‐6 and TNF‐α at 7 days and 14 days. Data are presented as the means ± SD. ns, no statistical significance. **p < 0.01 and ***p < 0.001. *n* = 3 per group.

To explore the effects of PEPGS hydrogels on immuneregulation in vivo, paraffin sections were immunostained for iNOS (red) and CD206 (green) as well as IL‐6 and TNF‐α. As shown in Figure 6A, immunofluorescence of iNOS and CD206 in the wound section was observed at 7 and 14 days. iNOS expression in the wound bed of the control group exhibited an increase compared to that in the PEPGS group at 7 and 14 days. CD206 expression in the wound bed of the PEPGS group increased progressively compared with the control group at 7 and 14 days. The iNOS/CD206 ratio of the PEPGS group at 7 days (0.20) and 14 days (0.22) were significantly lower than the ratio of the control group at 7 days (2.39) and 14 days (3.89; Figure 6B, *p* < 0.001 for both).

Immunohistochemical staining was used to assess IL‐6 and TNF‐α expression in the wound bed. The results showed that IL‐6 expression in the wound bed of the PEPGS group was reduced dramatically at 7 and 14 days compared to that in the control group (Figure 6C,J, *p* < 0.001). TNF‐α expression in the wound bed of the PEPGS group was also significantly reduced compared to that in the wound bed of the control group at both 7 and 14 days (Figure 6D,K, *p* < 0.001, *p* < 0.01).

Angiogenesis was assessed by immunofluorescence, immunohistochemistry and micro‐computed tomography (micro‐CT; Figure 6E–G). Immunofluorescence staining of CD31 and α‐SMA (Figure 6E) and immunohistochemical staining of CD31 (Figure 6F) revealed the numbers of newly formed and mature blood vessels in wounds of the different groups. Qualitative data showed denser blood vessels in the PEPGS group than in the control group at both 7 and 14 days (Figure 6H,I,L). Micro‐CT three‐dimensional reconstructed images revealed a substantially denser network of blood vessels in the wounds of the PEPGS group compared to the control group (Figure 6G). Quantification of the newly formed vessel area further confirmed these findings (Figure 6M, *p* < 0.001).

### 3.7 Proteomics analysis of the effect of PEPGS hydrogels on mitochondrial function during diabetic wound healing

**FIGURE 7:**
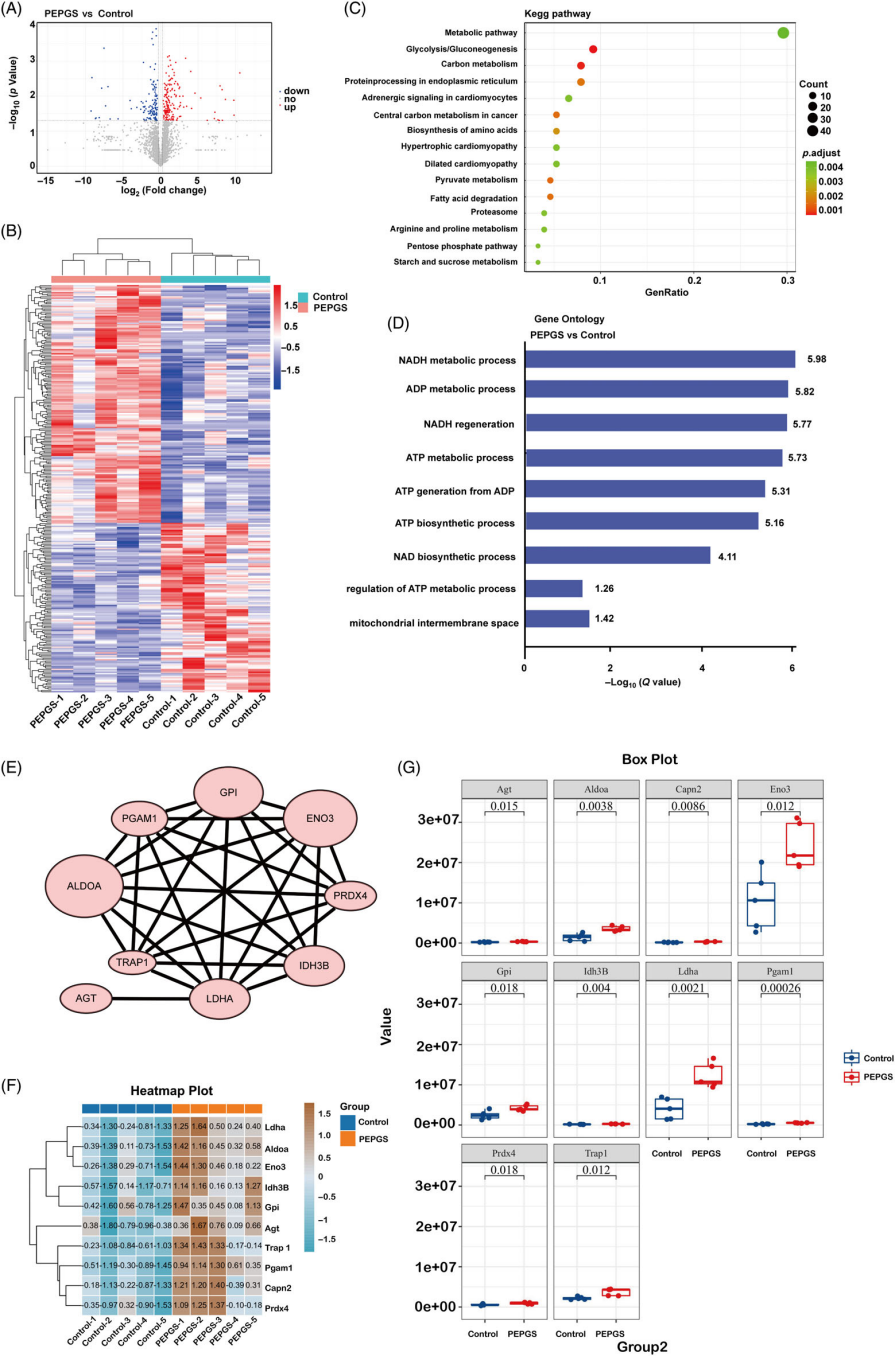
Label‐free proteomics analysis of the effect of poly (glycerol sebacate)‐co‐poly (ethylene glycol)‐co‐poly (propylene glycol) (PEPGS) hydrogel on mitochondrial metabolism. (A, B) Volcano plots and hierarchical cluster analysis showing the upregulated and downregulated proteins in response to PEPGS hydrogel treatment in the diabetic wound microenvironment. (C) The result of Kyoto Encyclopaedia of Genes and Genomes pathway enrichment analysis. (D) The result of Gene Ontology analysis. (E) Protein–protein interaction network of upregulated proteins involved in improved mitochondrial metabolism during diabetic wound healing. (F) The correlation analysis of upregulated proteins. (G) Quantitative results of upregulated proteins in the PEPGS hydrogel group compared with the control group (fold change ≥1.2, *p* < 0.05). *n* = 5 per group. NADH, nicotinamide adenine dinucleotide.

Label‐free proteomics analysis was performed on samples collected from wound tissue on day 14. Analysis of the results showed that 3349 expressed proteins were identified, and there were 171 upregulated and 122 downregulated proteins according to significant differences analysis, as shown in the volcano plots (Figure 7A). For differences between groups in different proteins, hierarchical cluster analysis was conducted for up‐ and down‐regulated protein sets between wound tissue from the control group and the PEPGS group (Figure 7B). Furthermore, the Kyoto Encyclopaedia of Genes and Genomes pathway enrichment analysis revealed that metabolic pathways were significantly enhanced in the PEPGS group (Figure 7C). To further explore the biological function of protein variations associated with PEPGS treatment of diabetic wounds, Gene Ontology was used for the analysis, which mainly included three main functional areas: molecular functions, cellular components and biological processes. The results revealed that a variety of protein molecules participated in a variety of processes of mitochondrial metabolism and ATP production (Figure 7D).

MRM is a germplasm in view of the target molecule spectrum analysis technology. MRM enables the quantitative determination of target proteins. We used MRM to quantitatively analyse the above target proteins, after analysis of the artificial proofreading exported indexed retention time internal standard peptides retention time data (Figure S1A). The results showed that in all cases the coefficient of variation (SD/mean) of the peptides was less than 20%, qualitative stability between repeated tests was good and the system error was small (Figure S1B). Functional protein association network analysis not only identified the central role of differentially expressed proteins but also demonstrated the important role of interacting with leading proteins to exert therapeutic effects and accelerate wound healing in response to PEPGS treatment (Figure 7E). Correlation analysis results of differential protein were shown in Figure 7F, which are consistent with the above Gene Ontology analysis (Figure 7D). Finally, the quantitative results showed that the expression of differentially‐expressed proteins was up‐regulated in the PEPGS group compared with that in the control group (Figure 7G, fold change ≥1.2, *p* < 0.05).

## 4 DISCUSSION

Macrophages play a crucial role in the inflammatory response by dynamically altering their phenotypes and functions in the progression of diabetic wounds.^42^, ^43^ Failure of the macrophage phenotypic switch from M1 to M2 is associated with impaired wound healing in diabetes.^44^ To further verify the type of macrophages in the two groups, immunofluorescence staining was performed to detect iNOS and CD206 in paraffin sections.^45^ These results showed that excessive inflammatory response was present in the diabetic wound. Impaired angiogenesis is one of the most striking features of diabetic chronic wounds, and has become one of the most serious clinical challenges.^46^ In our research, immunohistochemical staining of CD31 was used to assess the impaired angiogenesis in diabetic wounds. The qualitative results showed that the degree of impaired angiogenesis was significantly worse in the diabetic wounds (*p* < 0.01). MDA can be used as a marker to reflect the severity of oxidative damage in vivo.^47^ The antioxidant enzyme is the main defensive line that scavenges ROS, protects mitochondrial contents and stabilizes the mitochondrial membrane.^47^, ^48^, ^49^ SOD, CAT, and GSH‐Px are the main enzymes making up antioxidant systems.^47^ Therefore, the levels of the above indicators were measured to show that higher oxidative stress was present in the diabetic wounds. However, mitochondrial dysfunction due to electron transport chain impairment generally leads to decreased intracellular ATP production.^50^, ^51^ In this study, we took the levels of ATP production as mitochondria functions, results showed that reduced energy metabolism was present in diabetic wounds. To understand the correlation between mitochondrial functions and oxidative stress, inflammatory response and impaired angiogenesis in diabetic wounds, we identified ultimately that mitochondrial dysfunction is positively correlated with oxidative stress, inflammatory response and impaired angiogenesis in diabetic wounds.

Inspired by the above results, we originally developed a bioenergetically active PEPGS hydrogel based on enhanced mitochondrial metabolism to support energy production and thus improve endogenous cells biological behaviour and immunoregulation in diabetic wound healing. The PEPGS hydrogels use glycerol as the core, which is a metabolic substrate for restoring mitochondrial metabolism. In addition, they possess improved mechanical properties compared to PEGS hydrogels. Assessment of biocompatibility is important for biomaterials.^52^, ^53^ In our study, HUVECs and NIH 3T3 fibroblasts were chosen to assess the cytotoxicity of the PEPGS hydrogels in vitro. The observed results indicated that PEPGS hydrogels present a good growth‐promoting effect on HUVECs and NIH 3T3 cells, which is beneficial for further vascular and collagen tissue formation.

A recent study revealed that during late‐phase wound healing, type‐2 immune signals protect mitochondrial function by regulating and adjusting the mitochondrial stress response, which ultimately strengthens macrophage M2 polarization to repair the wound.^54^ M1 macrophages persist in the long term without transitioning to M2 macrophages in chronic diabetic wounds, which is considered to result in sustained inflammation and impaired tissue repair.^55^, ^56^ Our analytic results of diabetic wounds (Figure 1A,B) are consistent with this viewpoint. Thus adaptation in immune cell metabolism leads not only to changes in energy metabolism but also to regulation of the activation phenotype of immune cells.^57^ To confirm the ability of PEPGS to induce macrophage polarization, we selected some representative genes and analysed their expression by RT–qPCR and flow cytometry, results showed PEPGS hydrogels facilitated M2 macrophage polarization of RAW 264.7 macrophages.

Massive accumulation of ROS alters the mitochondrial membrane potential, which in turn leads to the energy crisis of cells in diabetic wounds, and even mediates ageing and apoptosis.^58^, ^59^ Therefore, the JC‐1 probe and Mito‐Tracker Red CMXRos were utilized to measure the ΔΨm of mitochondria.^47^ As the core of energy metabolism, mitochondria are involved in various biological processes.^49^ In the process of wound healing, energy metabolism (ATP production) provides power for cell migration, proliferation, angiogenesis, collagen formation and various physiological processes. New research suggests that during the late phase of wound healing, catabolic processes such as ATP production are activated by OXPHOS in macrophages.^54^ Glycerol is the most important substrate for the biosynthesis of G3P, which ultimately generates ATP during OXPHOS.^60^, ^61^ Consequently, we confirmed that PEPGS hydrogels could maintain ΔΨm, reduce ROS levels and improve ATP synthesis by restoring mitochondrial metabolism.

Within the diabetic hyperglycaemic environment, AGEs act as one of the major factors underlying the pathology of delayed healing of diabetic wounds.^7^ Dermal fibroblasts have a key role in maintaining and remodelling the skin ECM.^62^ They play a major role in the wound repair process, synthesizing collagen and elastin.^63^ Diabetic wound healing is delayed as a result of impairment of the biosynthetic or functional response of fibroblasts.^64^ Vascular lesions are also a cause of the inability of chronic diabetic wounds to heal.^65^ Hyperglycaemia leads to macrovascular and microvascular complications by damaging vascular endothelial cells, resulting in limb ischaemia and loss of sensation and eventually leading to the development of gangrene.^66^ PEPGS hydrogels possess remarkable biocompatibility and beneficial cell adhesion properties to aid cell proliferation, meanwhile also promoting more energy production by restoring mitochondrial metabolism. In our study, HUVECs and NIH 3T3 fibroblasts were chosen to investigate the biological effects of PEPGS hydrogels on the activities of the two cell types. These results suggested that PEPGS overcame the suppression of HUVECs and NIH 3T3 fibroblastic cells under a diabetic environment in vitro.

Hydrogel‐based biomaterials are widely used as wound dressings for the skin, as they maintain a moist wound environment, prevent tissue dehydration and facilitate cell proliferation, angiogenesis and collagen synthesis.^67^ In this study, we used full‐thickness wounds in STZ‐induced diabetic rats to evaluate the efficacy of PEPGS hydrogels in treating diabetic wounds. Finally, as demonstrated by a rat diabetic wound model in vivo, PEPGS hydrogels promoted the healing of rat diabetic wounds via their pro‐angiogenesis, pro‐fibrogenesis and immunoregulatory effects. In our study, proteomics analysis was used for global assessment of the regulation mechanism of PEPGS hydrogels on mitochondrial function during diabetic wound healing. Finally, these results showed that PEPGS is of great significance in aspects of the regulation of ATP and ATP metabolic processes, ATP generation from ADP, mitochondrial intermembrane space, ATP biosynthetic process, nicotinamide adenine dinucleotide metabolic process and regeneration and NAD biosynthetic process. In a word, proteomics analysis results suggested that PEPGS of the bioenergetically active potential was an improved treatment option for diabetic wounds.

## 5 CONCLUSIONS

Inspired by the results of clinical research, which showed that excessive inflammatory response, impaired angiogenesis and higher oxidative stress, together with reduced energy metabolism persisted during diabetic wound repair. We developed innovatively a bioenergetically active PEPGS hydrogel to restoring mitochondrial function and support energy production, overcome suppression of endogenous cells and regulate immune status during diabetic wound healing.

Our research purpose is to solve clinical problems from a novel mitochondrial metabolism perspective. This innovative strategy focuses on hydrogel biomaterials and highlights the restored role of PEPGS hydrogels in mitochondrial metabolism and immunomodulation during diabetic wound healing firstly. It brings an in‐depth insight into biomaterials integrated with cellular metabolism and provides a promising direction for exploring the translation to clinical applications.

## AUTHOR CONTRIBUTIONS

Hui Sun, Jinghuan Huang, Changfeng Zhu and Wei Zhang designed the experiments. Xin Qi, Jingyi Si, Bohao Yin and Xin Wang carried out in vitro experiments. Xin Qi, Changfeng Zhu and Hui Sun carried out in vivo animal experiments. Xin Qi, Chenjun Liu, Jingjing Huang, Changfeng Zhu and Jinghuan Huang prepared the manuscript. Wei Zhang provided overall intellectual guidance and was the principal investigator of this group.

## FUNDING INFORMATION

This work was supported by the Shanghai Top Academic Leaders Program of the Shanghai Science and Technology Commission (Grant No. 20XD1402600), the National Key Research and Development Program of China (Grant No. 2018YFC1105603), the National Natural Science Foundation of China (Grant Nos. 82372390, 22274029, 82202695, and 81501862), the Special Project of Clinical Research for Health Industry from Shanghai Health Committee (Grant No. 20214Y0323), the Science and Technology Commission of Shanghai Municipality (Grant No. 22ZR1412000), and the Haobo Medical Technology (Shanghai) Co.Ltd.

## CONFLICT OF INTEREST STATEMENT

The authors declare no conflict of interest.

## Supporting information


**Table S1.** Primers used for RT–qPCR of RAW 264.7 cells.
**Figure S1.** Target protein quantitative determination. (A) Analysis of the artificial proofreading exported iRT internal standard peptides RT data and (B) peptides CV (SD/mean).

## Data Availability

The data that support the findings of this study are available from the corresponding author upon reasonable request.
