# Supplementary material for: A bioenergetically‐active ploy (glycerol sebacate)‐based multiblock hydrogel improved diabetic wound healing through revitalizing mitochondrial metabolism
Source: Cell Prolif. 2024 Feb 13;57(7):e13613. doi: 10.1111/cpr.13613 (PMC11216945; doi:10.1111/cpr.13613)
Supplement: Supplementary file 1 — Table S1. Primers used for RT–qPCR of RAW 264.7 cells. Figure S1. Target protein quantitative determination. (A) Analysis of the artificial proofreading exported iRT internal standard peptides RT data and (B) peptides CV (SD/mean). [file CPR-57-e13613-s001.docx]

**Supporting Information**

**A Bioenergetically-active Ploy (glycerol sebacate)-based Multiblock Hydrogel Improved Diabetic Wound Healing through Revitalizing Mitochondrial Metabolism**

Table S1. Primers used for RT-qPCR of RAW 264.7 cells

| Genes | Primers Sequence (F, forward; R, reverse; 5’−3’) |
| --- | --- |
| IL-4 | F: CCATATCCACGGATGCGACA  R: AAGCCCGAAAGAGTCTCTGC |
| IL-10 | F: GAGAAGCATGGCCCAGAAATC |
|  | R: GAGAAATCGATGACAGCGCC |
| Arg-1 | F: TGCTCACACTGACATCAACAC |
|  | R: GAGAATCCTGGTACATCTGGG |
| IL-6 | F: CCCCAATTTCCAATGCTCTCC  R: CGCACTAGGTTTGCCGAGTA |
| iNOS | F: TTGACGCTCGGAACTGTA |
|  | R: GTTGGTGGCATAAAGTATGTG |
| TNF-α | F: TAGCCAGGAGGGAGAACAGA |
|  | R: CCAGTGAGTGAAAGGGACAGA |

|  |  |
| --- | --- |
|  |  |


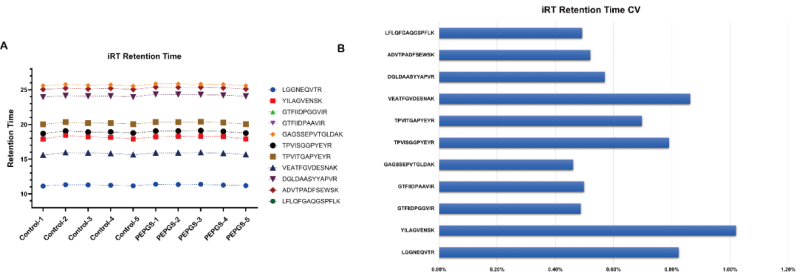


Figure S1. Target protein quantitative determination. A) Analysis of the artificial proofreading exported iRT internal standard peptides RT data and B) peptides CV (sd/mean).
